# D-glucaro-1,4-lactone alleviates acetaminophen-induced hepatotoxicity in mice via modulating gut microbiota and metabolites associated with *Lactobacillus*–glutamine and nicotinic acid pathways

**DOI:** 10.3389/fphar.2025.1627850

**Published:** 2025-07-09

**Authors:** Zhiying Song, Yiran Pan, Zeyu Wang, Yujing Chen, Yufeng Deng, Lele Wang, Qi Hu, Wenxiang Huang, Shuilin Sun, Baogang Xie

**Affiliations:** ^1^ Department of Infectious Diseases, The Second Affiliated Hospital of Nanchang University, Nanchang University, Nanchang, China; ^2^ Medical College of Jiaxing University, Key Laboratory of Medical Electronics and Digital Health of Zhejiang Province, Jiaxing University, Jiaxing, China; ^3^ Modern Industrial College of Traditional Chinese Medicine and Health, Lishui University, Lishui, China

**Keywords:** D-glucaro-1,4-lactone, acetaminophen, acute liver injury, *Lactobacillus*, glutamine, nicotinic acid

## Abstract

**Objective:**

This study investigated the hepatoprotective effect and underlying mechanisms of D-glucaro-1,4-lactone (1,4-GL), a natural compound found in fruits and vegetables, against acetaminophen (APAP)-induced acute liver injury (ALI) in mice, which had not been previously explored.

**Methods:**

A stable ALI model was established in male C57BL/6J mice using 300 mg/kg APAP after fasting. Mice were pretreated orally with glutathione (200 mg/kg), or 1,4-GL (100 mg/kg or 200 mg/kg) for five consecutive days before APAP challenge. Serum biochemical markers were measured. Liver histopathology was assessed via H&E staining. Gut microbiota composition was analyzed using 16S rRNA sequencing of fecal samples. Liver metabolites were profiled using ^1^HNMR metabolomics.

**Results:**

1,4-GL pretreatment (especially 200 mg/kg) significantly ameliorated APAP-induced liver damage: it reduced serum ALT, AST, TBIL, and MDA levels (*P* < 0.05), increased GSH and SOD levels (*P* < 0.05), and attenuated hepatic necrosis and inflammation. 1,4-GL increased the abundance of the beneficial gut bacterium Lactobacillus (significantly reduced by APAP) and elevated hepatic levels of protective metabolites isoleucine, glutamine, and nicotinic acid. Correlation analyses between gut microbiota and liver metabolites revealed that glutamine and nicotinic acid were significantly positively correlated with Firmicutes and Lactobacillus, while showing a significant negative correlation with Lachnoclostridium. Lactobacillus was identified as a key beneficial bacterium, whereas Lachnoclostridium was associated with increased disease severity.

**Conclusion:**

1,4-GL exerts a beneficial regulatory effect on APAP-induced ALI by the Lactobacillus-glutamine/nicotinic acid pathway, highlighting its potential as a therapeutic agent for drug-induced liver injury.

## 1 Introduction

Acetaminophen (APAP) is a commonly prescribed analgesic-antipyretic drug, typically dose-dependent as a hepatotoxin. Overdose of APAP is the most prevalent cause of drug-induced ALI and non-viral acute liver failure (ALF) (Stravitz and Lee, 2019). Over the years, in European and North American countries, APAP overdose has been the primary cause of ALI and ALF, and ALF related to APAP often progresses more rapidly and has a poorer prognosis compared to ALF caused by other etiologies (Larson et al., 2005; Reddy et al., 2016).

When administered at therapeutic doses, the majority of APAP undergoes glucuronidation and sulfation by phase II conjugation enzymes, converting it into non-toxic compounds that are subsequently excreted in the urine. Approximately 2% is excreted unchanged. Less than 10% of APAP is metabolized by cytochrome P450 enzymes (CYPs), primarily CYP2E1, into the highly reactive intermediate metabolite N-acetyl-p-benzoquinone imine (NAPQI). NAPQI is hepatotoxic but rapidly detoxified through conjugation with glutathione. However, upon APAP overdose, phase II metabolizing enzymes become saturated, and glutathione becomes insufficient to counteract the excessive production of NAPQI, leading to mitochondrial oxidative stress and dysfunction, ultimately resulting in hepatocyte necrosis (Lancaster et al., 2015; Jaeschke et al., 2012; Yang et al., 2018). Of course, exogenous supplementation of glutathione can enhance the liver’s detoxification capacity, alleviate APAP-induced hepatotoxicity, and thereby protect liver function (Mitchell et al., 1973; Masubuchi et al., 2011).

Liver physiological function is closely related to the stable intestinal microecology. Research indicates that the alterations in the composition of the gut microbiota play a significant role in the induction and progression of liver diseases (Wang et al., 2021; Solé et al., 2021). Meanwhile, disruptions in liver health status also can profoundly impact the gut microbiota, with more severe liver diseases leading to more significant alterations in gut microbiota (Zeng et al., 2020; Yao et al., 2021). Genomic sequencing of the gut microbiota suggests that in healthy conditions, the four dominant phyla in the gut are *Bacteroidetes*, *Firmicutes*, *Proteobacteria*, and *Actinobacteria* (Hugon et al., 2015). When liver diseases occur, the abundance of dominant phyla and beneficial bacterial genera within the gut will be affected and often tend toward an adverse progression (Bajaj, 2019). Supplementing key beneficial bacteria or intervening against major harmful bacteria will help delay the progression of liver disease and promote liver health (Albillos et al., 2020; Hsu and Schnabl, 2023).

As the largest digestive organ, the liver functions as the central hub for material metabolism in the body, participating in the metabolism of carbohydrates (Han et al., 2016), proteins (Bilsborough and Mann, 2006), and lipids (Nguyen et al., 2008). It is also the primary site for the metabolism of drugs and toxins, where toxic substances are metabolized into non-toxic or low-toxic forms and subsequently excreted from the body, thereby playing a crucial role in detoxification (Almazroo et al., 2017). The gut microbiota plays a significant role in maintaining organismal health (Hsu and Schnabl, 2023). The metabolic capacity of the gut microbiota is mainly associated with the composition of the microbiota and the stability of the gut microecology (Agus et al., 2021). The metabolites of the liver and intestine are tightly interconnected through the gut-liver axis. Therefore, correlation analysis of intestinal flora and altered metabolites in liver contributes to the understanding of the mechanism of action of drugs.

D-glucaro-1,4-lactone (1,4-GL) is a natural organic acid lactone found in fruits and vegetables such as apples, grapefruits, oranges, and cruciferous vegetables (Saluk-Juszczak, 2010). In recent years, 1,4-GL has garnered extensive attention in the medical field, with numerous studies indicating its ability to enhance antioxidant capacity (Saluk-Juszczak et al., 2008; Olas et al., 2007), positively regulate intestinal probiotics (Xie et al., 2014), and detoxify and inhibit the growth of cancer cells (Hanausek et al., 2003; Yang et al., 2018). However, to date, the potential beneficial effects of 1,4-GL on APAP - induced ALI has not been studied.

In this study, a stable APAP-induced ALI mice model was established and subsequently administered 1,4-GL orally. 16S rRNA sequencing of mice feces and Proton Nuclear Magnetic Resonance Spectroscopy (^1^H-NMR) metabolomics analysis of liver samples were performed to explore the mechanism of action of 1,4-GL.

## 2 Materials and methods

### 2.1 Reagents

APAP, glutathione, methanol (LC-MS), and methanol-d4 (CD3OD) were purchased from J&K Science Co., Ltd. (Beijing, China). 1,4-GL was purchased from Sigma-Aldrich (Shanghai, China). Sevoflurane for inhalation was purchased from Hengrui Pharmaceutical Co., Ltd. (Shanghai, China). Kits for measuring alanine aminotransferase (ALT), aspartate aminotransferase (AST), total bilirubin (TBIL), and reduced glutathione (GSH) (using the microplate method), total superoxide dismutase (SOD, using the WST-1 method), and malondialdehyde (MDA, using the TBA method) were all obtained from Nanjing Jiancheng Bioengineering Institute (Nanjing, China).

### 2.2 APAP-induced ALI mice and drug administration protocol

Male C57BL/6J mice (5–6 weeks old, 20 ± 2 g) were purchased from Zhejiang Weitong Lihua Experimental Animal Technology Co., Ltd. The mice were housed in the Specific Pathogen Free (SPF) animal laboratory of Jiaxing University (24°C ± 2°C, light turned on from 8:00 to 20:00, light/dark cycle for 12/12 h), given sufficient water and food during non-fasting periods. The use and care of animals in this study have been approved by the Experimental Animal Ethics Committee of Jiaxing University. The mice in all groups were euthanized by cervical dislocation after being anaesthetized with sevoflurane for inhalation.

To determine the optimal dosage for inducing a stable ALI model with APAP, 24 mice were randomly divided into four groups and administered with normal saline (0 mg/kg group) or 200, 300, and 400 mg/kg of APAP (dissolved in normal saline) via intragastric gavage after a 16-h fast (no water prohibition). After 24 h of gavage, blood samples were collected.

To evaluate the potential side effects of 1,4-GL on major organs, 12 mice were randomly divided into two groups as the Control (n = 6) and the 1,4-GL (n = 6) group. Mice in the Control group were gavaged with normal saline, while those in the 1,4-GL group were gavaged with 200 mg/kg of 1,4-GL (dissolved in normal saline) for five consecutive days. After that, blood samples and organs, including heart, liver, spleen, lung, and kidney, were collected from the mice. Furthermore, to investigate whether 1,4-GL pretreatment can mitigate APAP-induced hepatotoxicity, 42 mice were randomly divided into five groups: Control (n = 6), Model (n = 9), Glutathione (n = 9), a1,4-GL (n = 9), and b1,4-GL (n = 9). The Control and Model groups received normal saline, the Glutathione group received 200 mg/kg Glutathione, and the a1,4-GL and b1,4-GL groups received 100 mg/kg and 200 mg/kg 1,4-GL respectively (both dissolved in normal saline) for five consecutive days. After a 16-h fast (no water prohibition), all groups except the Control group received 300 mg/kg APAP via intragastric administration. One hour after APAP treatment, the corresponding groups were orally administered normal saline, Glutathione (200 mg/kg), or 1,4-GL (100 mg/kg or 200 mg/kg). Mice were returned to an *ad libitum* diet after 6 h. The modeling success was rigorously defined by following criteria: (1) Serum ALT and AST levels exceeding 200 U/L (vs. <50 U/L in controls), indicating hepatocellular damage; (2) ​**​** Hepatic centrilobular necrosis, inflammatory infiltration via H&E staining. Twenty-four hours after APAP treatment, the mice were sacrificed and the blood, liver and colorectal feces samples were collected for further assays.

### 2.3 Detection of serum biochemical indicators

The collected blood samples were centrifuged at 4,000 rpm for 10 min to separate the serum. A full-wavelength microplate reader (Thermo Fisher Scientific Oy, Finland) was used to measure the levels of serum ALT, AST, TBIL, GSH, SOD, and MDA, to assess the severity of liver injury and antioxidant capacity.

### 2.4 Hematoxylin-eosin (HE) staining of mice tissues

The heart, liver, spleen, lung, and kidney tissue samples of mice were taken and fixed with 4% paraformaldehyde for 24 h. Following fixation, the tissues were subjected to transparency, immersed in wax, and then embedded in paraffin. The embedded tissues were sliced into sections (4 μm, 10 sections per group), deparaffinized, and stained with HE. After dehydration and mounting, the slides were observed under an optical microscope.

### 2.5 16S rRNA sequencing analysis of fecal samples

Six colonic fecal samples were randomly selected in each group. The PowerMax Kit (MoBio Laboratories, Unites States) was used for extracting total DNA. The highly mutable V3-V4 region of the 16S sequence for PCR amplification was selected. After quality assessment of the amplification products using agarose gel electrophoresis, the products were purified and then quantify them using the PicoGreen dsDNA Kit (Invitrogen, Unites States) on the Illumina NovaSeq 6,000 sequencing platform.

After performing quality control, noise reduction, assembly, and chimera removal on the raw sequencing data, Amplicon Sequence Variants (ASVs) were obtained. These ASVs were then clustered and annotated. Species difference analysis and functional analysis were performed based on ASVs.

### 2.6 ^1^H-NMR metabolomics detection and analysis

After retrieving liver tissue from −80°C storage, 0.20 g was thawed at room temperature and placed into a tissue homogenizer. 50 μL of 2.0 M phosphate buffered saline (PBS) was added, followed by the gradual addition of a total of 2.0 mL of 80% methanol in two aliquots for homogenization and extraction. The tissue homogenates were vortexed for 30 s and ultrasonic treatment for 10 min 1.0 mL of the supernatant was transferred to a 1.5 mL centrifuge tube after centrifuging at 5,000 rpm for 10 min, which was concentrated and dried using a vacuum centrifugal concentrator (Jiaimu Technology Co., Ltd., Beijing, China) at 50°C. 550 μL of methanol-d4 was added to the dried sample for redissolution and transferred to a 5 mm NMR tube for ^1^H-NMR analysis in Bruker 600 MHz NMR spectrometer (Bruker, AVANCE NEO 600M, Germany).

Baseline correction, phase adjustment, and zero-point calibration of ^1^H-NMR spectral were performed using Topspin software (version 3.7.0, Bruker Biospin GmbH, Rheinstetten, Germany). Subsequently, the data were normalized, and residual water and methanol peaks were removed using MATLAB software (R2024a, MathWorks, Massachusetts, Unites States). The data were then imported into SIMCA software (version 14.1, Umetrics, Umea, Sweden) for Orthogonal Partial Least Squares-Discriminant Analysis (OPLS-DA). The metabolites in the liver tissue ^1^H-NMR spectral were identified using Chenomx NMR Suite software (version 8.2, Chenomx Inc., Edmonton, Canada) and concerning previously established metabolite standards (Yu et al., 2018). The intensity of metabolites were integrated using Matlab software, and subsequently, VIP values were calculated based on the OPLS-DA.

### 2.7 Statistical analysis

Statistical analysis of the data was conducted using Graphpad Prism version 9.5.1 (Graphpad Software, San Diego, CA, Unites States). Multiple comparisons were performed using one-way analysis of variance (ANOVA). Quantitative data were expressed as mean ± standard deviation (SD), and *p* < 0.05 was considered statistically significant.

## 3 Results

### 3.1 No side effects of 1,4-GL on the major organs of healthy mice

**FIGURE 1:**
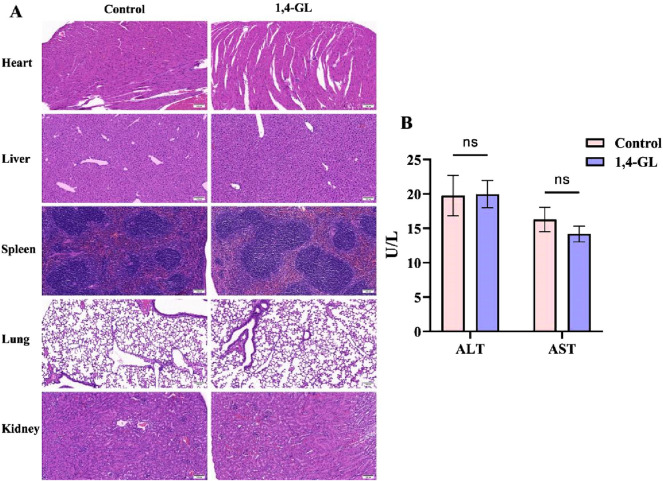
Compare the histopathology of tissues and the serum levels of ALT and AST between mice administered with 1,4-GL and healthy mice. **(A)** HE staining images of heart, liver, spleen, lung, and kidney tissues from healthy mice and healthy mice administered with 1,4-GL (HE, ×200). **(B)** Serum ALT and AST levels in healthy mice and healthy mice administered with 1,4-GL (“ns” represents no statistical difference).

The histopathology of the heart, liver, spleen, lung, and kidney in the 1,4-GL group was compared with that of healthy mice after 1.4-GL treatment (200 mg/kg). The pathological characteristics of the 1,4-GL group were similar to those of the Control group, including neatly arranged cardiomyocytes with pale red cytoplasm and blue-stained nuclei; intact hepatic lobules with orderly arranged hepatic plates; a clear demarcation between the red pulp and white pulp of the spleen, with dense lymphocytes in splenic follicles and no congestion in splenic sinuses; clear and intact alveolar structures without inflammatory infiltration; regularly shaped glomeruli and closely packed renal tubules (Figure 1A). The liver inflammation indexes ALT and AST were compared between the two groups of mice, and there was no significant difference between the two groups. 1,4-GL treatment did not cause the elevation of serum ALT and AST (Figure 1B).

### 3.2 ALI mice model was successfully induced by 300 mg/kg APAP

**FIGURE 2:**
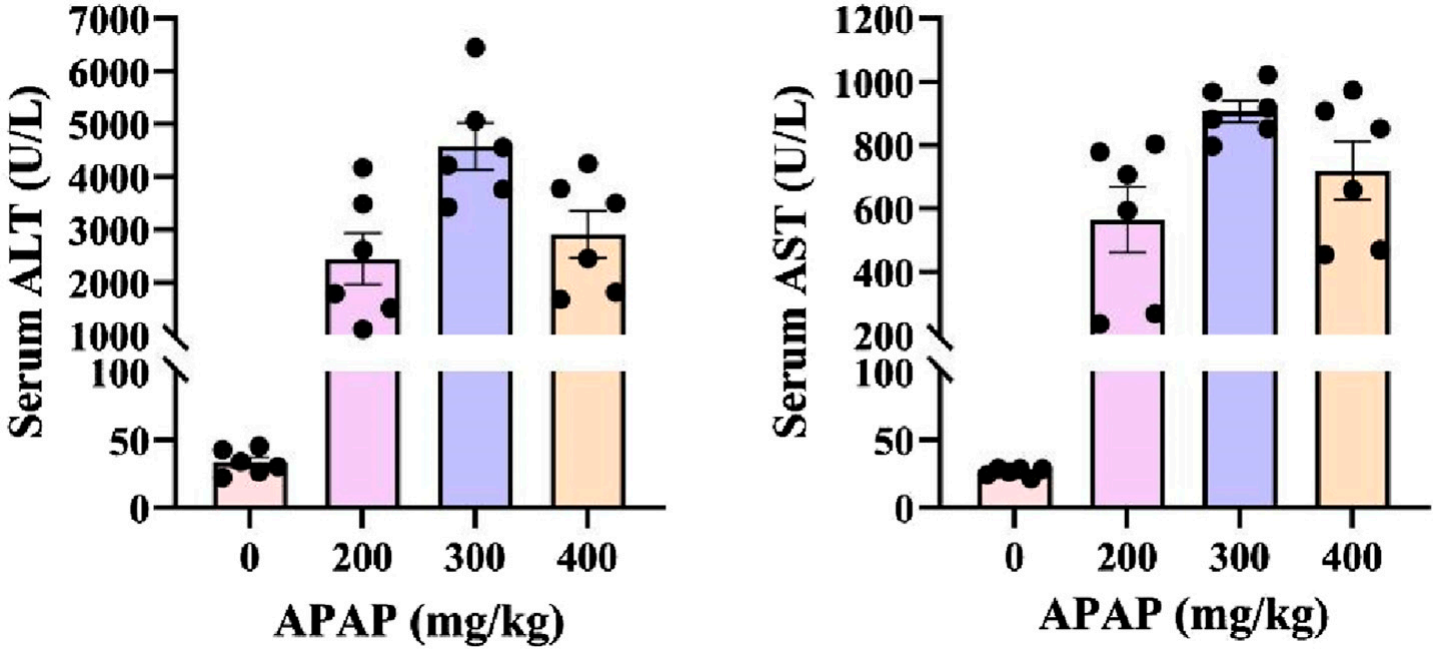
Serum ALT and AST levels of ALI mice induced by different APAP administration concentrations.

A comparison of serum ALT and AST levels was conducted between healthy C57BL/6J mice that were not took APAP and mice induced by APAP doses of 200 mg/kg, 300 mg/kg, and 400 mg/kg. Stable fluctuations of serum ALT or AST in healthy mice were observed. Among the three groups treated by different APAP doses, the serum ALT and AST values of the mice in the 300 mg/kg group exhibited most stable (Figure 2).

### 3.3 Significantly improved serum biochemical indexes of ALI mice pretreated with 1,4-GL

**FIGURE 3:**
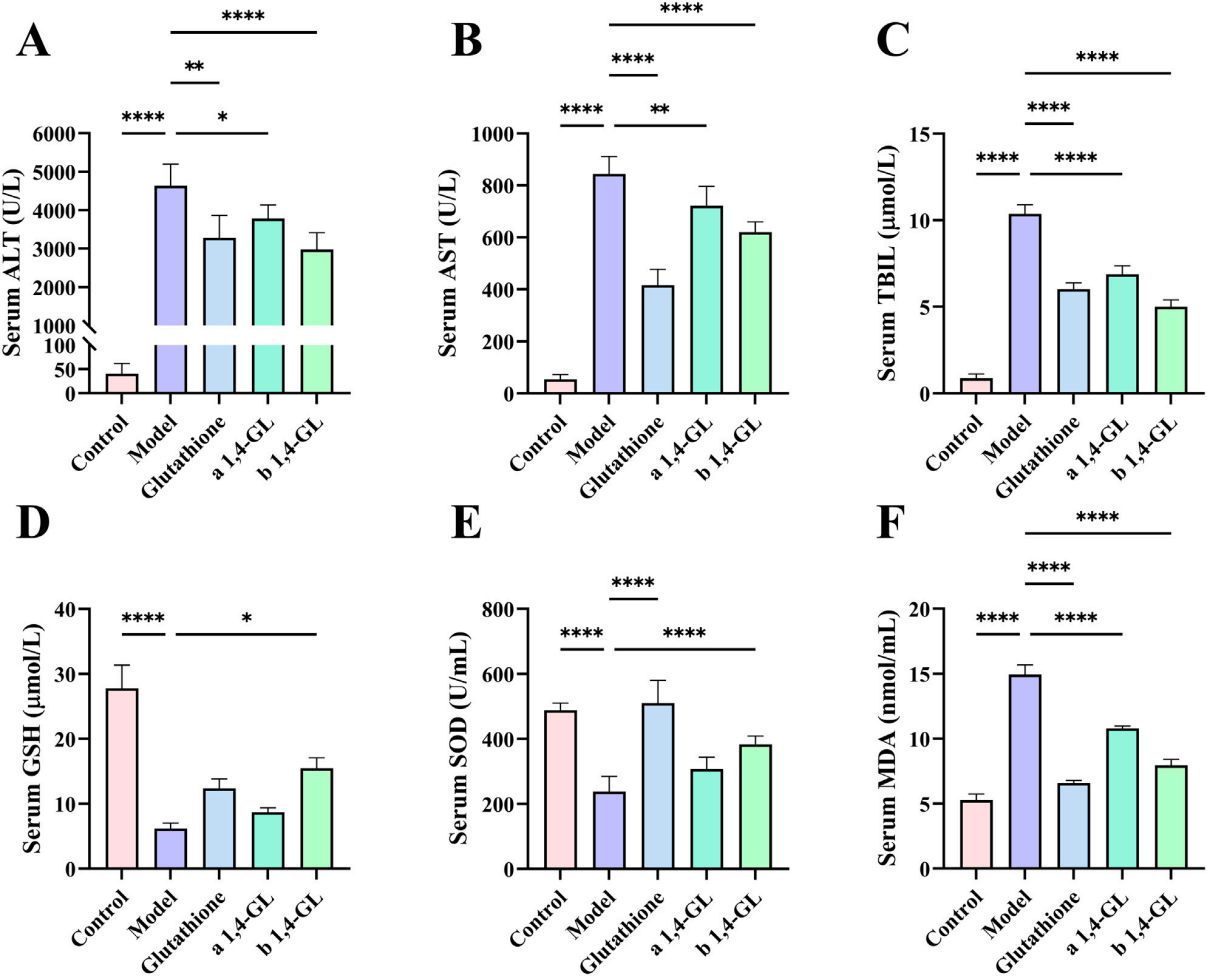
Serum levels of ALT, AST, TBIL, GSH, SOD, and MDA in mice in the Control, Model, Glutathione, a1,4-GL, and b1,4-GL groups. **(A)** Serum ALT level. **(B)** Serum AST level. **(C)** Serum TBIL level. **(D)** Serum GSH level. **(E)** Serum SOD level. **(F)** Serum MDA level (* indicates a statistically significant difference: *p < 0.05; **p < 0.01; ****p < 0.0001).

Compared to the Control group, the Model group of APAP induced ALI mice showed significantly elevated serum levels of ALT, AST, TBIL, and MDA, and showed a substantial depletion of GSH and SOD (Figures 3A–F). However, ALI mice pretreated with glutathione or 1,4-GL showed decreased serum levels of ALT, AST, TBIL, and MDA, and increased serum levels of GSH and SOD compared to the Model group (Figures 3A–F). The specific manifestations are as follows: compared with the Model group, the serum levels of ALT, AST, TBIL, and MDA in the Glutathione, a1,4-GL, and b1,4-GL groups were significantly reduced (*p* < 0.05, Figures 3A–C,F). The b1,4-GL group showed the most significant improvement in GSH level (*p* < 0.05, Figure 3D), and the SOD levels in the Glutathione and b1,4-GL groups were significantly elevated (*p* < 0.05, Figure 3E). What deserves more attention is that the serum ALT and TBIL levels in the b1,4-GL group mice were the lowest compared with those in the Model, Glutathione, and a1,4-GL groups, while the serum GSH level was the highest (Figures 3A,C,D).

### 3.4 1,4-GL treatment improved the pathological injury of liver tissue

**FIGURE 4:**
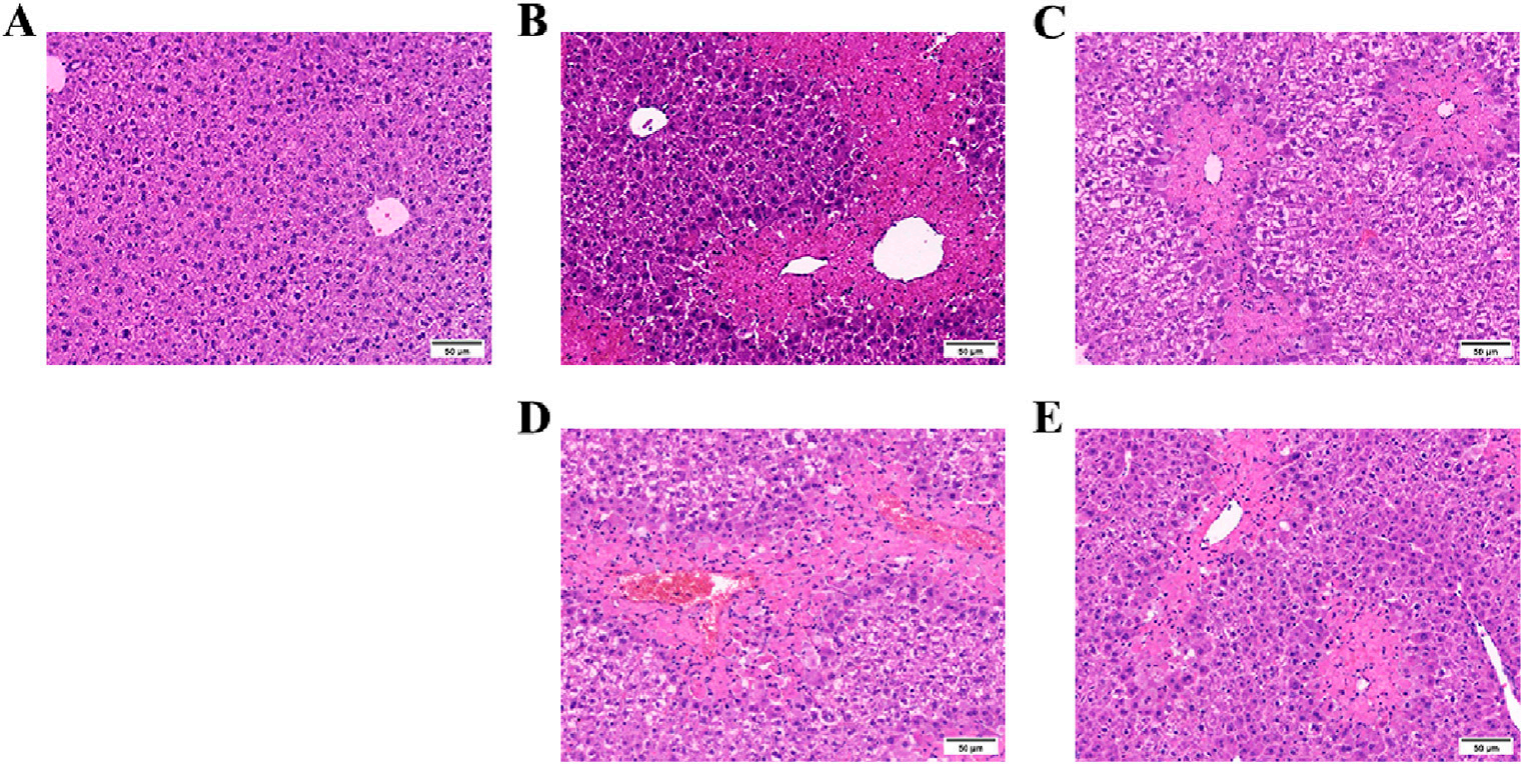
HE staining images of liver tissues of mice in the Control, Model, Glutathione, a1,4-GL, and b1,4-GL groups (HE, ×100). **(A)** The Control group. **(B)** The Model group. **(C)** The Glutathione group. **(D)** The a1,4-GL group. **(E)** The b1,4-GL group.

The liver histopathology of healthy mice in the Control group showed that the structure of the hepatic lobule was intact, the arrangement of hepatocytes and hepatic cords was regular and orderly, the degeneration and necrosis of hepatocytes were not observed, and the structure of hepatic sinuses was intact (Figure 4A). In the Model group, the hepatic lobules centered on the central vein showed map-like and large flake bleeding and necrosis, the structure of the hepatic lobules was blurred, the hepatic cord was disorganized, and inflammatory cells were infiltrated. Most of the hepatic cells showed obvious swelling and severe degeneration, some cells were dissolved, the nucleus was broken, the boundary between cells was unclear, and the hepatic sinuses were congested (Figure 4B). Liver of ALI mice treated with glutathione or 1,4-GL compared with the Model group: the necrotic area of liver tissue was significantly reduced, the structure of hepatic lobule was still preserved, the hepatic cord was disordered, and inflammatory cells were infiltrated. Mild to moderate swelling and watery degeneration were seen in most hepatocytes, some cells were dissolved, the boundary between cells was unclear, nuclear fragmentation was seen, and no obvious hepatic sinus congestion was observed (Figures 4C–E). Among them, the Glutathione and b1,4-GL groups had more obvious pathological improvement than the a1,4-GL group (Figures 4C,E).

### 3.5 16S rRNA sequencing of gut microbiota

**FIGURE 5:**
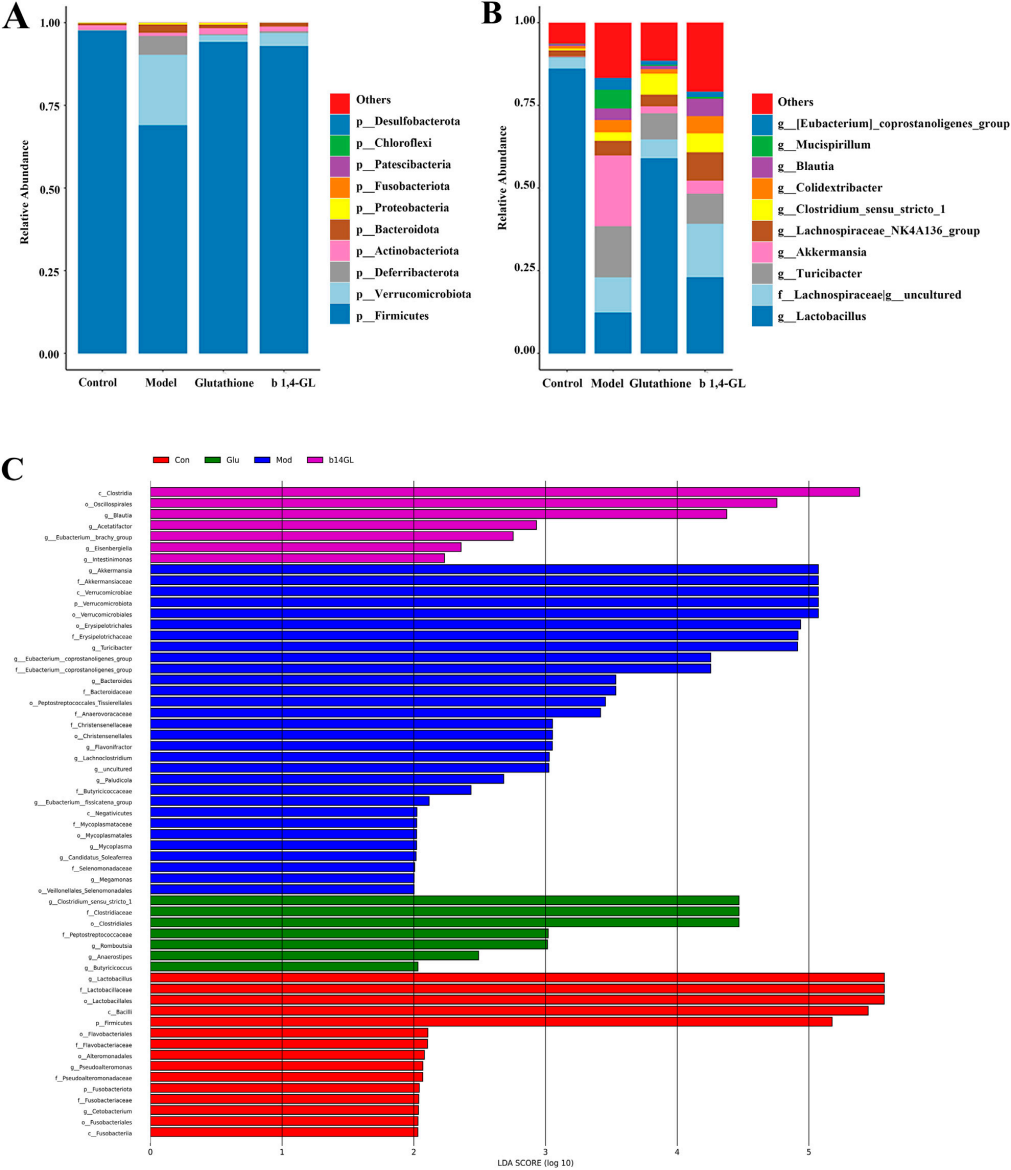
16S rRNA sequencing of fecal samples from mice in the Control, Model, Glutathione, and b1,4-GL groups. **(A)** The top 10 most abundant gut microbiota compositions at the phylum level across groups. **(B)** The top 10 most abundant gut microbiota compositions at the genus level across groups. **(C)** LEfSe analysis of gut microbiota across groups.

To examine the gut microbiota by 16S rRNA high-throughput sequencing, six mice in each group were randomly selected for collecting colon fecal samples in the b1,4-GL, Control, Model, and Glutathione groups. The gut microbial composition of each group was compared, and the gut microbial composition changed significantly at both the phylum and genus levels after APAP exposure (Figures 5A,B). At the phylum level, *Firmicutes*, *Actinobacteria*, *Bacteroidetes*, and *Proteobacteria* were the four dominant phyla in the gut microbiota of mice in the Control group, whereas the four dominant phyla in the Model group were *Firmicutes*, *Verrucomicrobia*, *Deferribacteres*, and *Bacteroidetes*. In the Glutathione and b1,4-GL groups, the dominant phyla were *Firmicutes*, *Verrucomicrobia*, *Actinobacteria*, and *Bacteroidetes*. At the genus level, the abundance of *Lactobacillus* was very high in the Control group, but it decreased in the other three groups exposed to APAP, with the lowest abundance in the Model group. Multiple genera in the Model group exhibited significant changes in abundance compared to the Control group, while the gut microbial composition of the Glutathione and b1,4-GL groups showed an intermediate state between the Control and Model groups.

The LDA Effect Size (LEfSe) analysis revealed that the Control group had 15 species, the Model group had 30 species, the Glutathione group had 7 species, and the b1,4-GL group had 7 species, suggesting significant differences in gut microbiota composition among groups. Compared with the Model, Glutathione and b1,4-GL groups, the abundance of *Lactobacillus* in the Control group had the most significant changes (Figure 5C).

### 3.6 ^1^H-NMR metabolomics analysis of liver tissue

**FIGURE 6:**
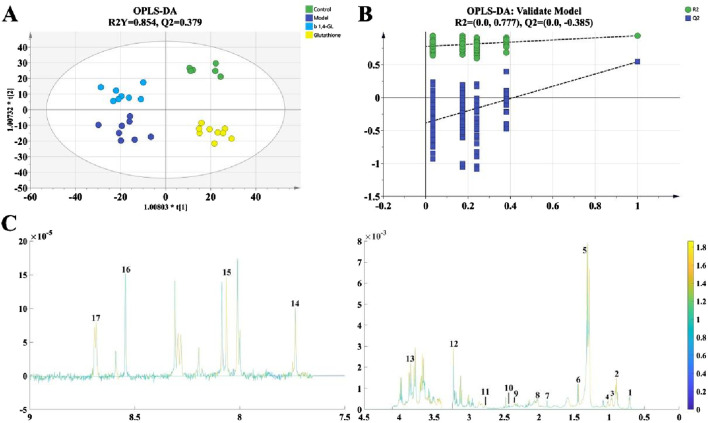
^1^H-NMR metabolomics analysis of liver tissue from mice in the Control, Model, Glutathione, and b1,4-GL groups. **(A)** OPLS-DA analysis based on seven-fold cross-validation method. **(B)** 200 permutation tests. **(C)** ^1^H-NMR spectrum of 1 mouse, with numbers 1-17 annotating the positions of 17 metabolites analyzed in this study on the spectrum. The specific metabolites corresponding to these numbers are listed in Table 1.

The metabolomics data of liver tissues based on ^1^H-NMR were analyzed and compared by Orthogonal Partial Least Squares-Discriminant Analysis (OPLS-DA). Based on seven-fold cross-validation method, the R^2^Y value was 0.854, and Q^2^ was 0.379. The variables for the Control, Model, Glutathione, and b1,4-GL groups were located in four distinct quadrants with clear boundaries and no overlap (Figure 6A). The Glutathione and b1,4-GL groups were positioned between the Control and Model groups, closer to the Control group compared to the Model group. After conducting 200 permutation tests, the R^2^ value was 0.777, and Q^2^ was −0.385, indicating no overfitting of the OPLS-DA model (Figure 6B). 17 metabolites were identified and their VIP values calculated by OPLS-DA were presented in Figure 6C. The metabolites with VIP value greater than 1 included leucine, isoleucine, acetate, glutamine, trimethylamine, choline, lysine, formate, and nicotinic acid. Among them, compared with the control group, the contents of isoleucine, glutamine, lysine, formate and nicotinic acid in the model group were significantly reduced, while the metabolite contents of glutathione and 1,4-GL treated mice were increased, and isoleucine, glutamine and nicotinic acid were significantly increased in the 1,4-GL group (Table 1).

**TABLE 1:** The relative contents of 17 metabolites in the Control, Model, Glutathione, and b1,4-GL groups.

NO	Metabolites	Control	Model	Glutathione	1,4-GL	VIP value
1	Bile acid	25.04 ± 9.38	28.67 ± 14.16	35.17 ± 9.32	27.52 ± 4.45	0.75
2	Lipid	66.87 ± 15.81	57.38 ± 25.17	78.25 ± 20.70	73.48 ± 26.68	0.49
3	Leucine	21.49 ± 7.56	21.11 ± 7.79	38.44 ± 8.76^a^	37.53 ± 12.66^a^	1.13
4	Isoleucine	12.87 ± 7.86^a^	6.07 ± 2.24	17.20 ± 4.51^a^	13.29 ± 3.06^a^	1.23
5	Lactate	1.97 ± 0.54	1.93 ± 0.52	2.55 ± 0.13^a^	2.29 ± 0.37	0.81
6	Alanine	125.38 ± 27.55	131.83 ± 31.04	132.00 ± 7.26	133.82 ± 10.80	0.41
7	Acetate	19.18 ± 6.39	22.72 ± 6.94	34.63 ± 4.66^a^	18.47 ± 3.47	1.42
8	N-Acetylglucosamine	132.73 ± 29.94	126.85 ± 36.55	155.24 ± 22.85	173.99 ± 50.09^a^	0.96
9	Glutamic acid	93.36 ± 28.78	65.36 ± 29.82	107.58 ± 12.35^a^	73.56 ± 10.36	0.95
10	Glutamine	39.26 ± 5.60^a^	18.52 ± 5.84	34.89 ± 9.08^a^	25.54 ± 6.33^a^	1.17
11	Trimethylamine	23.95 ± 4.63	23.35 ± 7.18	35.06 ± 3.43^a^	32.15 ± 8.99^a^	1.09
12	Choline	256.82 ± 73.34	188.84 ± 89.20	350.75 ± 27.78^a^	239.35 ± 20.84	1.02
13	Lysine	0.83 ± 0.03^a^	0.58 ± 0.17	0.77 ± 0.07	0.62 ± 0.17	1.07
14	Uridine	18.07 ± 1.82	16.63 ± 2.66	22.67 ± 5.55	17.48 ± 0.83	0.97
15	Hypoxanthine	12.16 ± 1.83	9.16 ± 4.20	13.54 ± 1.60	11.59 ± 1.88	0.98
16	Formate	12.73 ± 1.50^a^	7.53 ± 2.39	12.41 ± 1.53^a^	7.83 ± 1.20	1.28
17	Nicotinic acid	11.96 ± 3.37^a^	6.52 ± 1.78	12.75 ± 2.06^a^	9.16 ± 0.80^a^	1.02

^a^ indicates statistical significance when compared to the Model group, *p <* 0.05.

### 3.7 Correlation of liver metabolites and gut microbiota

**FIGURE 7:**
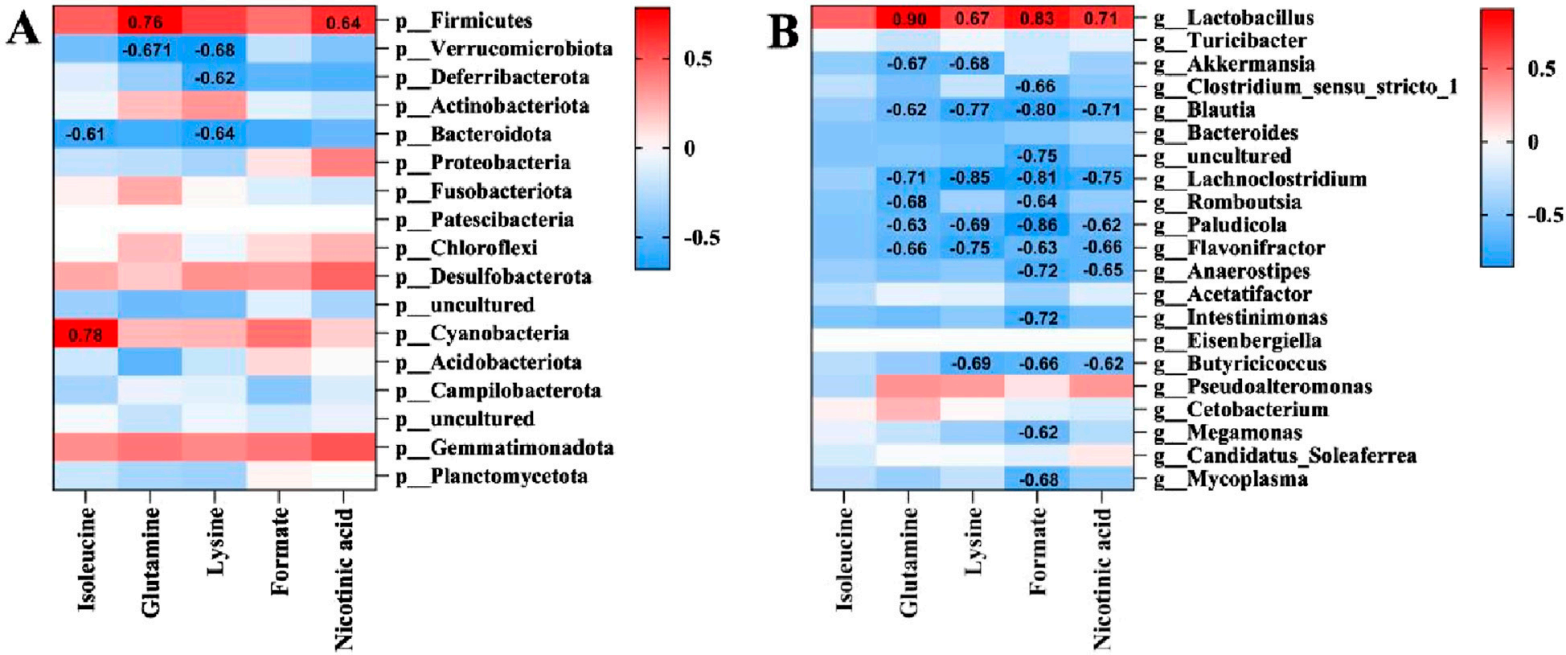
Correlation analysis between differential metabolites and differential gut microbiota. **(A)** Correlation analysis at the phylum level. **(B)** Correlation analysis at the genus level.

A correlation analysis between the liver metabolites, which are significantly affected by APAP hepatotoxicity such as isoleucine, glutamine, lysine, formate, and nicotinic acid, with the various bacterial phyla of the gut microbiota (Figure 7A), as well as the 21 differential bacterial genus identified through LEfSe analysis (Figure 7B) were performed. The results indicate that isoleucine is significantly positively correlated with *Cyanobacteria* (r = 0.78) and significantly negatively correlated with *Bacteroidota* (r = −0.61). Glutamine and nicotinic acid show significant positive correlations with *Firmicutes* (r = 0.76, 0.64) and *Lactobacillus* (r = 0.90, 0.71), while they exhibit the most significant negative correlation with *Lachnoclostridium* (r = −0.71, −0.75). Glutamine and lysine display the most notable negative correlation with *Verrucomicrobiota* (r = −0.67, −0.68). Additionally, lysine and formate also demonstrate a high positive correlation with *Lactobacillus* (r = 0.67, 0.83) and a significant negative correlation with *Lachnoclostridium* (r = −0.85, −0.81).

The absolute values of the correlation coefficient r values between the aforementioned liver metabolites and gut microbiota are all greater than 0.6, indicating a moderate to high degree of linear correlation. Among them, the most noteworthy findings are the significant positive correlations of glutamine and nicotinic acid with *Firmicutes* and *Lactobacillus*, as well as their significant negative correlation with *Lachnoclostridium*. These results correspond with the LEfSe analysis, where the abundances of *Firmicutes* and *Lactobacillus* in the Control group were significantly higher than those in other groups, while *Lachnoclostridium* was a significantly increased differential bacterial genus in the Model group (Figure 5C).

## 4 Discussion

Our findings indicated that in mice induced by 300 mg/kg of APAP, the levels of serum ALT, AST, and TBIL (which represent the severity of liver injury) were significantly higher than those in healthy mice. Notably, there was obvious necrosis in the liver tissues, suggesting that excessive APAP can cause marked liver damage in mice within a short period of time. 1,4-GL, a natural compound found in fruits and vegetables, has garnered considerable attention in the medical community due to its antioxidant, detoxifying, anti-tumor, and intestinal flora-regulating properties. In this study, mice orally administered with 1,4-GL exhibited normal pathological structures in their major organs and had serum ALT and AST levels within the normal range, when compared with healthy mice. This indicated that 1,4-GL has no significant adverse effects on the major organs, particularly no toxic or adverse effects on the liver.

Administering 1,4-GL to mice with APAP-induced ALI led to notable improvements in serum biochemical indicators, particularly at a dose of 200 mg/kg, and it effectively lowered serum ALT, AST, and TBIL levels. Therefore, 1,4-GL mitigated the hepatic cell damage caused by APAP. 1,4-GL has commendable antioxidant properties similar to glutathione, and it significantly increased SOD levels while decreasing MDA levels. Moreover, when compared to direct exogenous glutathione supplementation, 1,4-GL supplementation resulted in a more significant increase in serum GSH levels in ALI mice, and this characteristic likely plays a crucial detoxifying role in mitigating APAP - induced liver toxicity.

The sequencing results from the Control group of mice indicated that the gut microbiota composition of this group was within the normal range, with a stable gut microenvironment. Both the gut and liver were in a healthy state. It is noteworthy that, compared to the Control group, the Model group exhibited the most significant decrease in the abundance of *Firmicutes* and *Lactobacillus*, while the gut microbiota changes of ALI mice treated with glutathione and 1,4-GL were improved to some extent. *Lactobacillus*, belonging to the *Firmicutes* phylum, is a probiotic crucial for maintaining the overall gut microecological balance (Rastogi and Singh, 2022). In this study, it was found that the hepatotoxicity of APAP led to a significant decrease in the abundance of *Lactobacillus*. Jeon-Kyung Kim’s research team discovered that supplementing with *Lactobacillus reuteri* could increase the degradation of APAP (Kim et al., 2018). Bejan J Saeedi’s team also confirmed that supplementation with *Lactobacillus rhamnosus* could attenuate the oxidative damage caused by APAP on *Drosophila* and liver damage caused by APAP on C57BL/6 mice (Saeedi et al., 2020). It can thus be inferred that the severity of liver damage caused by the hepatotoxicity of APAP is closely associated with the abundance of *Lactobacillus*. In this study, the Model group exhibited the lowest abundance of *Lactobacillus* and the most severe liver damage. The abundance of *Lactobacillus* increased in ALI mice after supplementing with 1,4-GL, which is consistent with our team’s previous finding in hypercholesterolemic mice where 1,4-GL elevated the abundance of *Lactobacillus* (Xie et al., 2014). And the Vijendra Mishra team has confirmed the antioxidant potential of *Lactobacillus* in reducing oxidative damage, enhancing oxygen radical scavenging capacity, and boosting antioxidant enzyme activity (Mishra et al., 2015). Therefore, we consider the increased abundance of *Lactobacillus* as one of the significant reasons for the substantial improvement in APAP-induced hepatotoxicity by 1,4-GL.

The ALI mice showed significantly lower contents of liver metabolites isoleucine, glutamine, lysine, formate, and nicotinic acid than healthy ones. Among them, isoleucine, as a branched-chain amino acid, improves liver function by enhancing hepatic metabolism and detoxification capabilities, as well as providing a nitrogen source for protein synthesis (Olde Damink et al., 2007; Kawaguchi et al., 2013). Additionally, isoleucine can also provide carbon source for glutamine synthesis (Holeček, 2024), while glutamine can enhance liver detoxification ability (Brusilow et al., 2010; Watford, 2000), improve intestinal mucosal barrier function (Rao and Samak, 2012), and enhance the antioxidant ability of scavenging oxygen free radicals (Evens et al., 2004), and also is the main substrate for liver, kidney and intestinal gluconeogenesis (Holeček, 2024). The essential amino acid lysine possesses reducing properties, and its primary site of metabolism is the liver (Matthews, 2020; Zhang et al., 2023). Formate mainly originates from the metabolism of the gut microbiota (Hughes et al., 2017), with the liver being the primary site for formate metabolism. Formate is an intermediate metabolite in one-carbon metabolism, closely associated with embryonic development and neurological diseases (Washburn et al., 2015; Pai et al., 2015; Pietzke et al., 2020). And nicotinic acid is an important coenzyme that is abundant in liver and involved in cellular redox reactions, which can enhance the antioxidant capacity of liver and promote liver fat metabolism (Tupe et al., 2011; Li et al., 2014). Hepatotoxicity of APAP led to a decrease in the contents of these metabolites, and the corresponding function might be impaired. It is noteworthy that supplementation with 1,4-GL had mitigated the reduction in the contents of these metabolites to a certain extent and significantly elevated the contents of isoleucine and glutamine, which possess liver detoxification capabilities, as well as nicotinic acid, which has antioxidant properties. Therefore, we speculate that the protective effect of 1,4-GL against APAP-induced ALI is closely related to the increased content of these metabolites.

By analyzing the correlation between gut microbiota and liver metabolites, we found that the *Lactobacillus* and *Lachnoclostridium*, which belong to *Firmicutes*, deserve special attention. Only glutamine and nicotinic acid exhibited significant correlations with both *Firmicutes*, *Lactobacillus*, and *Lachnoclostridium*. *Lactobacillus*, as a crucial intestinal probiotic, not only plays a role in protecting the intestinal barrier function and maintaining intestinal health, but multiple studies have also shown that increasing *Lactobacillus* abundance is an effective means to alleviate liver injury (Huang et al., 2023; Fan et al., 2023; Saeedi et al., 2020). Compared to *Lactobacillus*, *Lachnoclostridium* plays a more complex role in the gut microbiota. *Lachnoclostridium* has been found to play an important role in improving non-alcoholic fatty liver disease (Ding et al., 2022; Dai et al., 2023), but also plays a crucial role in promoting atherosclerosis (Cai et al., 2022), and has been further identified as significantly enriched in colorectal adenocarcinoma patients and nasopharyngeal carcinoma patients with poor prognosis (Liang et al., 2020; Yu et al., 2024). Currently, there are few studies exploring the role of *Lachnoclostridium* in the progression of ALI, but an increase in *Lachnoclostridium* abundance has been observed in patients with severe intrahepatic cholestasis of pregnancy (Li et al., 2023). In this study, the abundance of *Lachnoclostridium* was significantly increased in the Model group, indicating that *Lachnoclostridium* is associated with severe ALI conditions.

**FIGURE 8:**
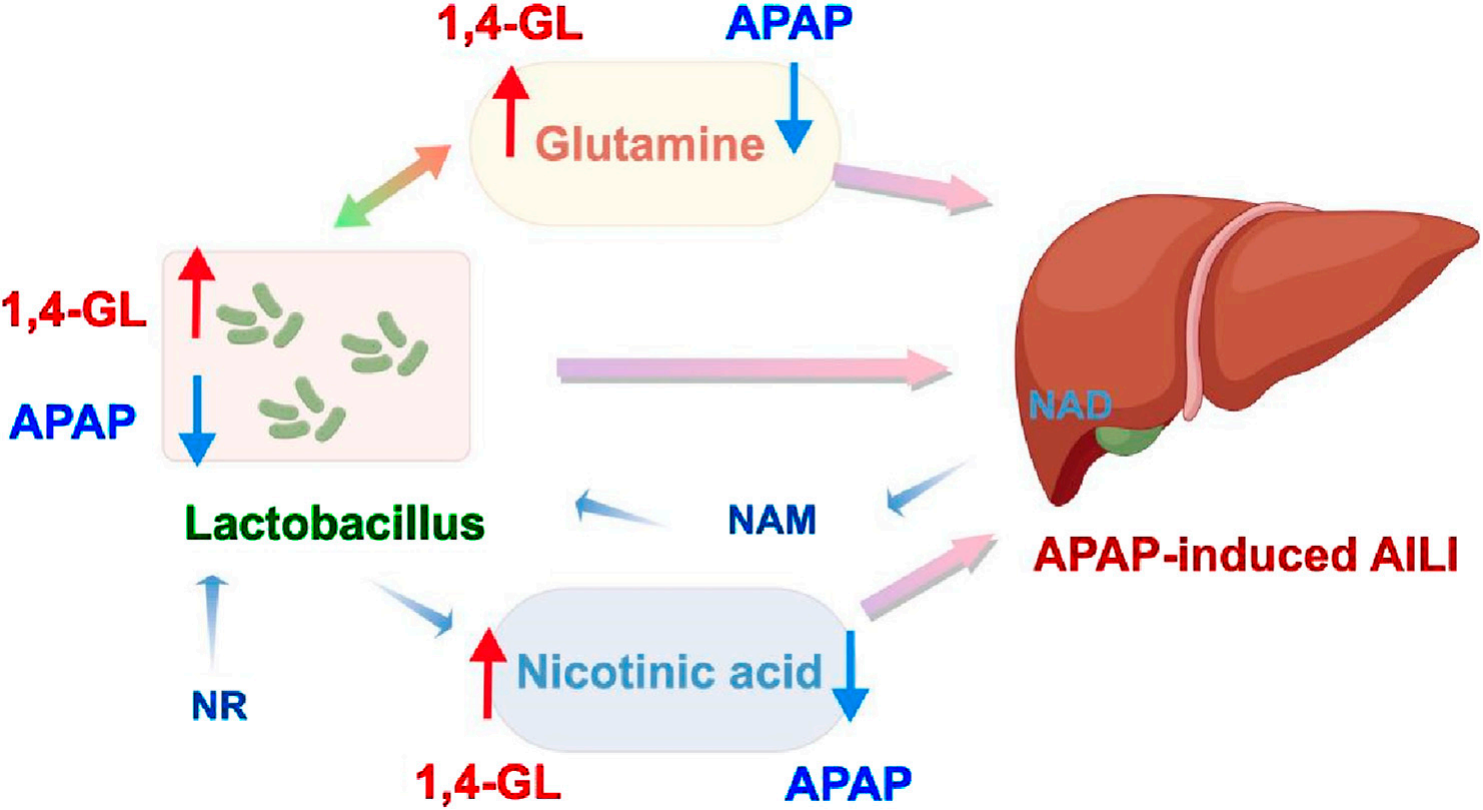
1,4-GL exerts its protective effect against APAP-induced ALI by regulating the *Lactobacillus*-glutamine, nicotinic acid-liver protection pathway.

Compared to *Lachnoclostridium*, the relationship between *Lactobacillus* and metabolites is more clearly defined. Glutamine can enhance the acid tolerance of *Lactobacillus*, thereby increasing its survival rate in acidic environments and promoting its growth and reproduction within the intestinal ecosystem (Teixeira et al., 2014). Glutamine’s protective effect on the intestinal mucosal barrier provides a stable intestinal environment for *Lactobacillus* (Rao and Samak, 2012). Studies have also found that supplementing with *Lactobacillus* can increase the absorption of glutamine into the blood (Zhu et al., 2024). Furthermore, dietary supplementation with glutamine can enhance the populations of *Lactobacillus* in the digestive tract of weaning piglets (Jiang et al., 2024). Therefore, there exists a mutually promoting relationship between glutamine content and *Lactobacillus* abundance, while they exhibit synergistic effects in maintaining liver health. Unlike glutamine, nicotinic acid is synthesized by the transformation of intestinal microbiota such as *Lactobacillus*. Intestinal microbiota converts host tissue-derived nicotinamide (NAM) and exogenically supplemented nicotinamide riboside (NR) into nicotinic acid into liver tissue, and nicotinic acid and its derivative nicotinamide adenine dinucleotide (NAD) in liver tissue play an antioxidant role in liver protection (Chellappa et al., 2022). In the process of nicotinic acid mitigating liver injury, *Lactobacillus* mainly plays the role of converting and synthesizing nicotinic acid. Both *Lactobacillus* and these two metabolites exhibit protective effects on the liver, while there exist interrelations between the *Lactobacillus* and metabolites. As a natural organic acid lactone, previous research demonstrated 1,4-GL can exert direct antioxidant activity, detoxification, and modulation of gut microbiota (Saluk-Juszczak et al., 2008; Xie et al., 2014; Hanausek et al., 2003). However, our study is the first to explore the hepatoprotective effect of 1,4-GL on APAP-induced ALI. Through 16S gut microbiota sequencing combined with ^1^H-NMR metabolomics analysis,we identified the key gut bacterial genera and liver metabolites that play a role in liver protection. These findings *suggest* that 1,4-GL’s hepatoprotection is linked to modulation of the *Lactobacillus*–glutamine/nicotinic acid axis (Figure 8), germ-free mice and glutamine/nicotinic acid supplementation to validate causality will be used in ongoing work.

Although the results achieved are encouraging and confirm the hepatoprotective effect of 1,4-GL, this study still has some limitations. Firstly, our study identified *Lactobacillus* as a key genus, future metagenomic sequencing or strain-specific qPCR could delineate species responsible for the observed effects. Additionally, the potential impact of intervening in these gut bacterial genera and/or metabolites on APAP-induced ALI, such as supplementing with *Lactobacillus* and/or glutamine, has not been explored. Thirdly, this study used male mice to avoid confounding effects of estrogen on APAP metabolism (e.g., CYP2E1 activity). Future work will include both sexes and larger cohorts to fully understand the hepatoprotective effects of 1,4-GL.

## 5 Conclusion

The study showed that 1,4-GL could significantly ameliorate APAP-induced ALI by regulating the disrupted gut ecological balance and increasing the abundance of *Lactobacillus*. Furthermore, 1,4-GL notably elevated the levels of isoleucine, glutamine, and nicotinic acid in the liver, thereby reducing liver injury. Correlation analysis revealed that *Lactobacillus* is a key beneficial bacterium, which is highly positively correlated with glutamine and nicotinic acid. Our findings indicated that 1,4-GL exhibits significant hepatoprotective effects on ALI through regulating the levels of *Lactobacillus*, glutamine and nicotinic acid, which may contribute to the prevention and therapy of ALI by regulating the *Lactobacillus*-metabolite-liver pathway.

## Data Availability

The original contributions presented in the study are publicly available. This data can be found here: https://www.ncbi.nlm.nih.gov/sra/PRJNA1284826.
